# Behavioral phenotypes of temporal lobe epilepsy

**DOI:** 10.1002/epi4.12488

**Published:** 2021-05-05

**Authors:** Bruce P. Hermann, Aaron F. Struck, Kevin Dabbs, Mike Seidenberg, Jana E. Jones

**Affiliations:** ^1^ Department of Neurology University of Wisconsin School of Medicine and Public Health Madison WI USA; ^2^ Department of Neurology William S Middleton Veterans Administration Hospital Madison WI USA; ^3^ Department of Psychology Rosalind Franklin University of Science and Medicine North Chicago IL USA

**Keywords:** behavior, phenotypes, psychopathology, temporal lobe epilepsy

## Abstract

**Objective:**

To identity phenotypes of self‐reported symptoms of psychopathology and their correlates in patients with temporal lobe epilepsy (TLE).

**Method:**

96 patients with TLE and 82 controls were administered the Symptom Checklist 90‐Revised (SCL‐90‐R) to characterize emotional‐behavioral status. The nine symptom scales of the SCL‐90‐R were analyzed by unsupervised machine learning techniques to identify latent TLE groups. Identified clusters were contrasted to controls to characterize their association with sociodemographic, clinical epilepsy, neuropsychological, psychiatric, and neuroimaging factors.

**Results:**

TLE patients as a group exhibited significantly higher (abnormal) scores across all SCL‐90‐R scales compared to controls. However, cluster analysis identified three latent groups: (1) unimpaired with no scale elevations compared to controls (Cluster 1, 42% of TLE patients), (2) mild‐to‐moderate symptomatology characterized by significant elevations across several SCL‐90‐R scales compared to controls (Cluster 2, 35% of TLE patients), and (3) marked symptomatology with significant elevations across all scales compared to controls and the other TLE phenotype groups (Cluster 3, 23% of TLE patients). There were significant associations between cluster membership and demographic (education), clinical epilepsy (perceived seizure severity, bitemporal lobe seizure onset), and neuropsychological status (intelligence, memory, executive function), but with minimal structural neuroimaging correlates. Concurrent validity of the behavioral phenotype grouping was demonstrated through association with psychiatric (current and lifetime‐to‐date DSM IV Axis 1 disorders and current treatment) and quality‐of‐life variables.

**Significance:**

Symptoms of psychopathology in patients with TLE are characterized by a series of discrete phenotypes with accompanying sociodemographic, cognitive, and clinical correlates. Similar to cognition in TLE, machine learning approaches suggest a developing taxonomy of the comorbidities of epilepsy.

**Key Points**

- We examined whether distinct behavioral phenotypes could be identified in patients with temporal lobe epilepsy.
- The nine SCL‐90‐R behavior problem scales were subjected to hierarchical clustering analytics.
- Three behavioral phenotype groups were identified: normal (42% of sample), mildly abnormal (35%), and globally impaired (23%).
- Behavioral phenotypes had orderly differences in cognitive and psychiatric features with modest clinical and imaging characteristics.
- Behavioral phenotypes and their correlates may offer new insights into the long‐standing problem of neuropsychiatric comorbidities of epilepsy.

## 1 INTRODUCTION

Population‐based investigations have demonstrated the increased prevalence of somatic,^1^ cognitive,^2^ and psychiatric^3^, ^4^ comorbidities of the epilepsies which are costly,^5^ increase healthcare utilization,^6^ and decrease quality of life.^7^, ^8^ Neurobehavioral complications may be even more prevalent in patients presenting for care at specialized medical centers, but considerable heterogeneity in their presence and severity has been demonstrated. By way of example, cognitive abnormalities are an especially problematic complication of temporal lobe epilepsy (TLE) and its neuropsychological profile is now known to be heterogeneous and reflected in reproducible cognitive phenotypes. These phenotypes range from (a) one typically expected and characterized by abnormal anterograde memory (that may be accompanied by concomitant language and/or executive dysfunction), to (b) less expected phenotypes including one characterized by intact cognition comparable to healthy controls, to (c) one demonstrating an unexpected global impairment across all tested cognitive domains.^9^, ^10^, ^11^, ^12^, ^13^, ^14^

Neuroimaging research has shown concomitant abnormalities consistent with the presence and degree of impairment across the cognitive phenotypes reflected in brain structure, connectivity (diffusion and resting state fMRI), and large‐scale covariance analyses of cortical/subcortical gray and white matter—the findings overall consistent with the hypothesis that disrupted networks rather than focal pathology primarily underlie the heterogeneous cognitive presentations of TLE.^11^, ^15^, ^16^, ^17^, ^18^

The issue of heterogeneity in the psychiatric complications of the epilepsies and a related taxonomy of psychopathological phenotypes has been investigated less often. In youth with new and recent onset epilepsies using a standard parent‐completed rating scale (Child Behavior Checklist), three behavioral phenotypes were identified that ranged from comparable to normally developing youth, to an intermediate group characterized by nonexternalizing behavioral problems (eg depression, anxiety), to a generally behaviorally disrupted group of clinical significance. These behavioral phenotypes were associated with diverse sociodemographic, clinical epilepsy, and neuroimaging correlates.^17^ In adults with focal epilepsies, distinct symptom‐based phenotypes of depression along with their accompanying cognitive complications have been identified.^19^

To date, a comparable approach has not been undertaken in regard to the behavioral complications of adults with epilepsy which is the purpose here. Using a common patient‐completed questionnaire of emotional‐behavioral distress, the aims of this investigation were to: (1) apply unsupervised machine learning analytics to identify underlying latent groups reflective of varying risk and patterns of psychopathology in patients with TLE, (2) characterize the correlates of the identified latent groups including sociodemographic (age, gender, education, handedness), clinical epilepsy (age of onset, duration, lifetime generalized seizures, medications, laterality), neuropsychological (intelligence, memory, executive function), and structural imaging characteristics (cortical thickness and volume and subcortical volumes), and (3) address the concurrent validity of the behavioral taxonomy via comparison to structured psychiatric interview indicators of past and current psychiatric history and treatment, self‐reported depression symptoms, and patient‐reported health‐related quality of life.

## 2 METHODS

### 2.1 Participants

Epilepsy patients were recruited from the epilepsy clinics of two collaborating medical facilities in the state of Wisconsin (University of Wisconsin‐Madison and Marshfield Clinic). Epilepsy clinic records were first screened by IRB‐approved project staff to preliminarily identify TLE patients who appeared to meet inclusion criteria. Those records were subsequently independently reviewed by project epileptologists to confirm eligibility (see below) after which the potential participants were contacted, informed of the project, invited to participate, and asked to consider identifying a spouse, relative, or friend who might serve as a control participant. The final participating sample was comprised of 94 individuals with TLE and 82 healthy controls.

More specifically, the selection criteria for the participants with epilepsy included the following: (a) chronological age between 18 and 63 years, (b) not intellectually impaired per history or prior cognitive testing (later confirmed by administration of the Weschler Adult Intelligence Scale‐3rd Edition (WAIS‐III) IQ >69), (c) complex partial seizures of definite or probable temporal lobe origin based on consensus conference review by epileptologists blinded to cognitive, behavioral, and research imaging findings, (d) no MRI abnormalities other than atrophy on clinical interpretation, and (e) no other neurological disorder. The consensus clinical review included all available interictal and/or continuous video/EEG monitoring, clinical semiology, clinical neuroimaging, and developmental and neurological history.

Initial selection criteria for the controls included the following: (a) chronological age between 18 and 63, (b) not intellectually impaired per history or prior cognitive testing (later confirmed by administration of the Weschler Adult Intelligence Scale‐ 3rd Edition (WAIS‐III) IQ >69), (c) either a friend, relative, or spouse of the participant with epilepsy, (d) no current substance abuse, or medical or psychiatric condition that could affect cognitive functioning, and (e) no episode of loss of consciousness greater than five minutes, identified developmental learning disorder, or repetition of a grade in school. The purpose of criterion “e” was to exclude control participants who suffered serious head injuries, demonstrated early and persistent cognitive/learning problems, or school failure, all of which could adversely impact cognition and result in a nonrepresentative healthy comparison group.

This project was reviewed and approved by the University of Wisconsin School of Medicine and Public Health Institutional Review Board, and all participants were informed of the nature and purposes of this investigation, their questions were answered, and signed informed consent was obtained. As will be described below, demographic, anamnestic, and clinical data were collected through a combination of direct clinical interview with patient and medical record review including records requested from other treating facilities.

### 2.2 Procedures

#### 2.2.1 Behavioral measures

The Symptom Checklist‐90 Item Revised (SCL‐90‐R) was administered to all TLE and control participants. The SCL‐90‐R is a 90‐item self‐report inventory designed to reflect the psychological symptom patterns of community, medical, and psychiatric respondents.^20^ Each item is rated on a five‐point scale of distress ranging from “not at all” to “extremely” and is scored across nine primary symptom dimensions. The SCL‐90‐R is an established instrument and commonly used in epilepsy research.^21^, ^22^, ^23^, ^24^ The internal consistency coefficient rating ranges from 0.90 for Depression to 0.77 for Psychoticism.^25^ A brief summary of the SCL‐90‐R scales, their abbreviations, and item content follows below.

##### Somatization

Item content assesses complaints arising from perceptions of bodily dysfunction with complaints focusing on cardiovascular, gastrointestinal, respiratory, and other systems with strong autonomic mediation. Items also cover pain and discomfort of the gross musculature and additional somatic equivalents of anxiety.

##### Obsessive‐compulsive

Item content assesses thoughts, impulses, and actions that are experienced as unremitting and irresistible and that are of an ego alien or unwanted nature. Behavior and experiences of a more general cognitive performance deficit are also included.

##### Interpersonal sensitivity

Item content assesses self‐deprecation, self‐doubt, and marked discomfort during interpersonal interactions, self‐consciousness, and negative expectations concerning interpersonal behavior with others and others’ perceptions of them.

##### Depression

Item content assesses dysphoric mood and affect, withdrawal of life interest, lack of motivation and energy, feelings of hopelessness, thoughts of suicide, and other cognitive, and somatic correlates of depression.

##### Anxiety

Item content assesses nervousness, tension, and trembling, as are panic attacks and feelings of terror, apprehension, and dread. Somatic correlates of anxiety are also assessed.

##### Hostility

Item content assesses thoughts, feelings, or actions that are characteristic of the negative affect state of anger. Items include the three modes of expression and reflect aggression, irritability, rage, and resentment.

##### Phobic anxiety

Item content assesses persistent fear responses to a specific person, place, object, or situation that is irrational and disproportionate to the stimulus and leads to avoidance or escape behavior. Items focus on the more pathognomonic and disruptive manifestations of phobic behavior.

##### Paranoid ideation

Item content assesses the cardinal characteristics of projective thought, hostility, suspiciousness, grandiosity, centrality, fear of loss of autonomy, and delusions.

##### Psychoticism

Item content assesses behaviors that reflect a withdrawn, isolated, and schizoid lifestyle along with first‐rank symptoms of schizophrenia including hallucinations and thought control. Item content assesses a gradual continuum ranging from mild interpersonal alienation to frank psychosis.

The analyses focused on these nine specific behavior scales, excluding the composite scales which are summary reflections of the specific scales.

#### 2.2.2 Clinical and sociodemographic interview

Medical records were reviewed and participants were interviewed to determine core epilepsy characteristics (age of recurrent seizure onset, duration of epilepsy, time since last seizure, estimated number of lifetime secondarily generalized seizures, seizure onset laterality, number of antiseizure medications [ASM]) and sociodemographic characteristics (age, gender, education, handedness, marital status, employment status, current financial aid).

#### 2.2.3 Neuropsychological assessment

TLE and control participants underwent cognitive assessment with focus on metrics of intelligence (WAIS‐III Verbal, Performance, and Full Scale IQ),^26^ memory (Wechsler Memory Scale‐III auditory and visual immediate and delayed recall),^27^ and executive function (Wisconsin Card Sorting Test [64‐card] perseverative responses,^28^ Stroop Interference,^29^ WMS‐III working memory index, and Trail Making Test‐B^30^, ^31^). This controlled longitudinal cohort investigation maintained use of the Wechsler Adult Intelligence Test‐Third Edition. Given the high correlations between the 3rd and 4th editions (eg, Full Scale IQ correlation of 0.94),^32^ the use of the older version is likely of minimal clinical significance.

#### 2.2.4 Self‐report measures

TLE patients completed measures of health‐related quality of life (QOLIE‐89),^33^ perceived seizure severity (Liverpool Seizure Severity Scale),^34^, ^35^ and the presence and severity of depressive symptoms (Beck Depression Inventory‐II).^36^

#### 2.2.5 Psychiatric diagnostic interview

Each control and epilepsy participant participated in an independent semistructured psychiatric interview using the Structured Clinical Interview (SCID) for DSM‐IV‐TR Axis I disorders,^37^ by a trained PhD psychologist. Variables of interest included the presence of lifetime‐to‐date and current psychiatric diagnoses, current mood disorder, current mental health treatment with medications, and psychiatric history in first‐degree relatives.

#### 2.2.6 MRI acquisition and processing

Images were obtained on a 1.5T GE Signa MRI scanner (GE Healthcare). Sequences acquired for each participant were T1‐weighted, three‐dimensional (3D) spoiled gradients recall (SPGR) using the following parameters: TE = 5ms, TR = 24ms, flip angle = 40 degrees, NEX = 1, slice thickness = 1.5 mm, slices = 124, plane = coronal, field of view (FOV) = 200 mm, and matrix = 256 × 256. All MR images were inspected before image processing. Image quality was rated from 0 to 4 (with 4 being the highest quality images) scale, and we required a minimum quality score of 3 for the scan to be included in the analysis. Images were transferred to a Mac OSX (Apple Inc) computer for processing with the FreeSurfer Software Suite. FreeSurfer is a known collection of tools for analyzing cortical and subcortical anatomy, and the program can be freely downloaded from (https://surfer.nmr.mgh.harvard.edu). The T1‐weighted MRI scans were used for cortical reconstruction and volumetric segmentation; details can be found in prior publications.^38^

The SCL‐90‐R, SCID, neuropsychological, and imaging data were all collected concurrently during the participants’ 2‐day study visit.

### 2.3 Data analyses

#### 2.3.1 Cluster analysis

Standardized T‐scores from the nine SCL‐90‐R scales were used for hierarchical clustering among patients with epilepsy. The optimal clustering method was determined by maximizing agglomerative coefficient by comparing “average,” “single,” “complete,” and “Ward” linkages (“cluster” 2.1.0 R package “Finding Groups in Data”: Cluster Analysis Extended.^39^ Next, the number of clusters was determined using the gap statistic. The optimal number of clusters was determined by finding the number of clusters in which the gap statistic was maximized (number of clusters limited between 2 and 5),^40^ using the method proposed by Tibshirani et al^40^ such that the cluster number is the lowest cluster number within 1 standard error of the local maximum. After an optimal number of clusters was determined, hierarchical cluster bootstrapping with replacement for 1000 trials was used to ensure stability of clustering. Final partitions were determined by the frequency of concurrence over the 1000 trials (“fpc”—Flexible Procedures for Clustering” 2.2‐3, Christian Henning R package). All statistical analysis was performed in R version 3.6.1.

#### 2.3.2 Clinical, demographic, and psychiatric analytics

First, group comparisons across the SCL‐90‐R scales were conducted by analysis of variance (ANOVA for epilepsy vs controls) or multivariate ANOVA (MANOVA for comparison of epilepsy phenotypes and controls) with Sidak correction for multiple comparisons. Targeted group comparisons were conducted for other continuous variables including neuropsychological test scores, continuous sociodemographic characteristics (eg, age), and patients' ratings of depression symptoms, quality of life, and seizure severity. Second, chi‐squared was used to examine dichotomous variables (eg, handedness, gender, lifetime‐to‐date and current Axis 1 psychiatric disorder, number of ASMs (mono vs polytherapy), ictal EEG onset laterality, history of mental health treatment, and family psychiatric history).

#### 2.3.3 Image analytics

The neuroimaging correlates of cluster membership focused on differences in cortical thickness and volume and subcortical (amygdala, caudate, hippocampus, thalamus) and cerebellum volumes between controls and behavioral phenotype groups using surface‐based group analyses with FreeSurfer's statistical tool, Qdec. Surface data were smoothed to improve intersubject averaging with a 15‐mm full width at half maximum Gaussian kernel. Age and gender (cortical thickness) as well as ICV (cortical and subcortical volumes) were modeled as covariates. To correct for multiple comparisons, a Monte Carlo simulation was implemented with an initial cluster‐forming threshold set to *P* <.05. Clusters were tested against an empirical null distribution of maximum cluster size built using synthesized Z‐distributed data across 10,000 permutations, producing clusterwise *P*‐values fully corrected for multiple comparisons. Subcortical volumes of interest (amygdala, hippocampus, thalamus, caudate) were compared with ICV and age as covariates.

## 3 RESULTS

The optimal agglomerative coefficient was found using the Ward method (0.949) and was used for hierarchical clustering. Other methods results were average (0.706), single (0.436), and complete (0.862). The optimal number of clusters determined by the Gap Statistic method was 3 (dendogram and cluster plot provided in Figures S1 and S2).

Table 1 provides information regarding the baseline characteristics of the control and overall TLE groups. Columns 2 and 3 show that participants with TLE had a significantly lower Full Scale IQ (FSIQ) than controls (*t*(180) = 5.94, *P* < .001), although still within the average range, with no other statistically significant differences. Columns 4 through 6 provide details of the behavioral phenotype groups and contrasts will be detailed below.

**TABLE 1:** Demographic and clinical epilepsy characteristics

	Controls (n = 82)	All TLE (n = 94)	TLE Cluster 1 (n = 39)	TLE Cluster 2 (n = 33)	TLE Cluster 3 (n = 22)
Age^a^	33.8 (12.4)	37.0 (11.7)	36.1 (12.5)	37.6 (11.3)	37.9 (11.2)
Education^a^	13.5 (2.4)	12.98 (2.4)	13.9 (2.4)	12.9 (2.2)	11.5 (1.9)
Gender M/F	37/51	29/65	12/27	12/21	5/17
Handedness^b^ L/R/UK	10/78/0	11/81/2	4/34/1	4/28/1	3/19/0
Onset age^a^		14.8 (10.8)	15.6 (12.2)	14.4 (9.3)	15.1 (10.99)
Duration of epilepsy^a^		22.1 (11.9)	20.1 (12.6)	22.7 (10.9)	22.7 (12.3)
AEDs (mean)		1.8	1.65	2.0	1.67
Full Scale IQ^c^	104.8 (16.7)	93.4 (15.7)	103.4 (18.0)	92.9 (18.8)	83.8 (7.6)

Note

Abbreviations: F, female; M, male.

^a^ Age, education, onset age, and duration of epilepsy are depicted in years with standard deviation in parentheses.

^b^ Handedness represented by L=left, R=right, UK=unknown.

^c^ Intelligence assessed by the Wechsler Adult Intelligence Scale‐III.

### 3.1 TLE vs controls: SCL‐90‐R

**FIGURE 1:**
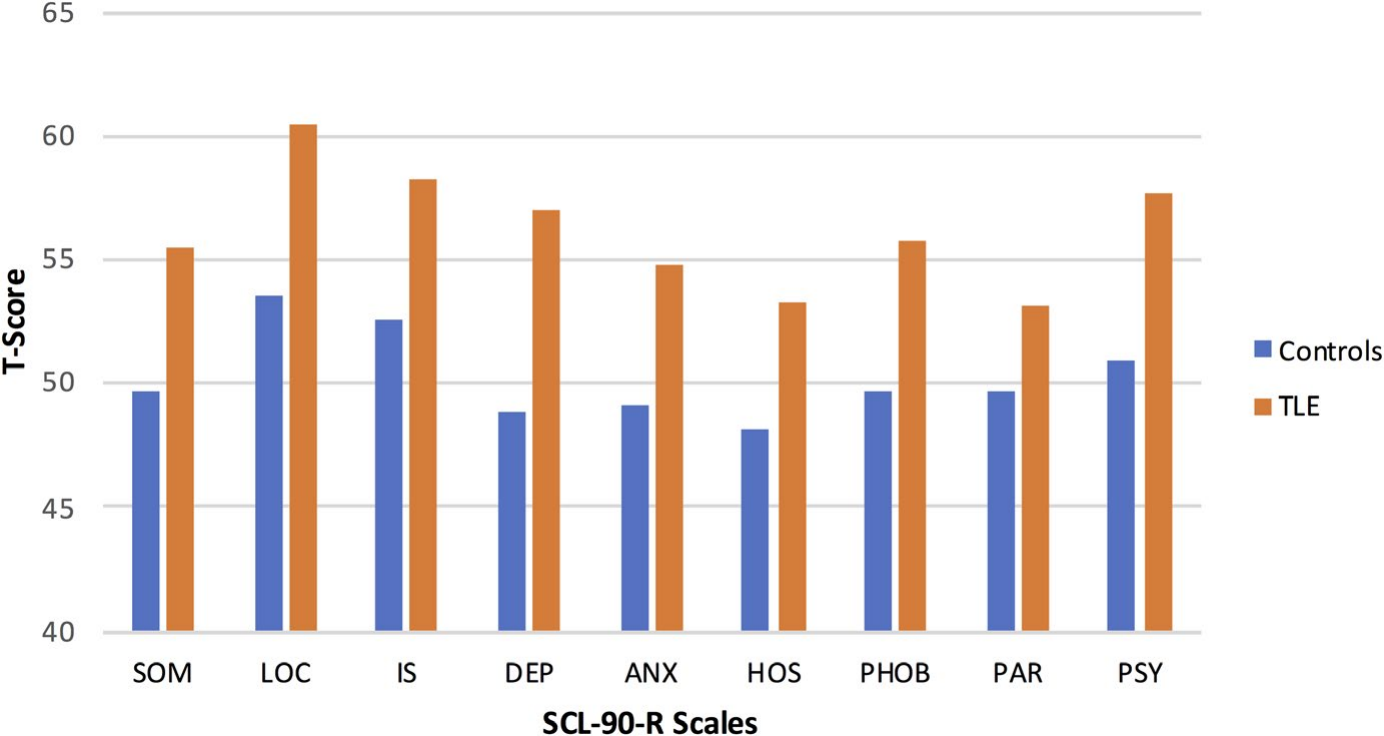
Mean SCL‐90‐R Scale Scores for Control and TLE groups. ANX, Anxiety; DEP, Depression; HOS, Hostility; IS, Interpersonal sensitivity; LOC, Obsessive‐compulsive; PAR, Paranoid ideation; PHOB, Phobic anxiety; Som, Somatization

MANOVA yielded a significant effect of group (TLE vs controls), Hotelling's T = 0.25, F = 4.5, *df*=9,165, *P* < .001, with significant univariate effects across all SCL‐90‐R scales (all *P*'s < .05) indicating significantly higher (worse) emotional‐behavioral status for the TLE group across all SCL‐90‐R scales (Figure 1).

### 3.2 TLE SCL‐90‐R clusters

**FIGURE 2:**
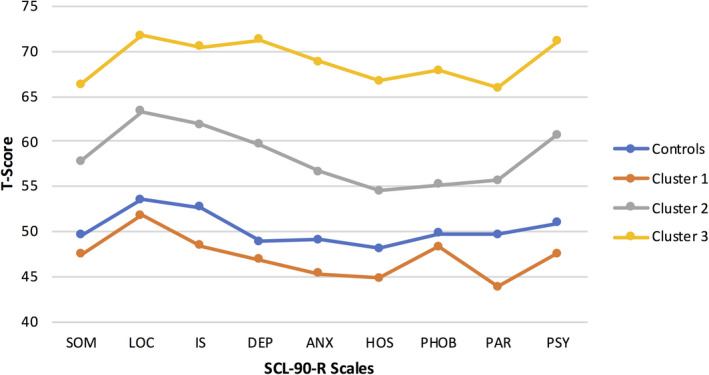
Control and TLE behavioral phenotype profiles. ANX, Anxiety; DEP, Depression; HOS, Hostility; IS, Interpersonal sensitivity; LOC, Obsessive‐compulsive; PAR, Paranoid ideation; PHOB, Phobic anxiety; Som, Somatization

MANOVA revealed a significant effect of cluster, Hotelling's T = 2.07, F = 12.4, *df*=27,485, *P *< .001, with significant univariate effects of cluster membership across all scales (all *P*'s < .001). Figure 2 provides a depiction of the mean SCL‐90‐R scores for the control and TLE cluster groups. Controls (blue) hovered near the mean T‐score of 50 (average) across all scales.

Cluster 1 (42% of TLE group) was not elevated compared to the controls on any SCL‐90‐R scale and was significantly lower (less distressed) than controls on the LIS (*P *= .005), ANX (*P* = .014), HOS (*P *= .023), PAR (*P *< .001), and PSY (*P* = 027) scales. Cluster 2 (35% of TLE patients) exhibited significant elevations compared to controls as well as Cluster 1 across all SCL‐90‐R scales (all *P*'s < .001‐.001). Four scale scores (LOC, IS, DEP, and PSY) were at or exceeded the 95th percentile. Cluster 3 (23% of patients) was the most abnormal group, scoring above controls as well as Clusters 1 and 2 across all SCL‐90‐R scales (all *P*'s < 0.001). All scale scores exceeded the 95th percentile with four scales (LOC, IS, DEP, and PSY) ≥98th percentile. Overall, there was a very orderly stepwise increase in self‐reported psychopathology across the TLE clusters ranging from no (Cluster 1) to marked (Cluster 3) elevations.

### 3.3 Neuropsychological status

Performance on measures of intelligence, memory, and executive function are summarized in Table 2.

**TABLE 2:** Intellectual, memory, and executive function performances

	Controls	Cluster 1	Cluster 2	Cluster 3	Univariate
Verbal IQ*	102.24 (15.9)^bc^	100.96 16.7)^de^	91.9 (17.1)^bd^	82.4 (7.3)^ce^	*P* <.001
Performance IQ*	107.7 (16.7)^bc^	105.3 (17.8)^de^	95.7 (17.9)^bd^	88.1 (10.8)^ce^	*P* <.001
Full Scale IQ*	104.8 (16.7)^bc^	103.4 (18.0)^de^	92.9 (16.8)^bd^	83.8 (7.6)^ce^	*P* <.001
Auditory Immediate**	107.7 (15.8)^bc^	105.9 (15.3)^de^	100.5 (15.2)^bd^	90.9 (15.5)^ce^	*P* =.004
Auditory Delayed**	110.55 (14.3)^abc^	101.8 (12.8)^ade^	101.4 (13.6)^bdf^	88.6 (17.8)^cef^	*P* <.001
Visual Immediate**	102.1 (15.9)^abc^	95.1 (11.9)^ad^	90.7 (12.5)^bd^	85.6 (14.5)^c^	*P* <.001
Visual Delayed**	102.8 (14.8)^abc^	94.6 (12.5)^ade^	94.1 (17.2)^bd^	86.4 (14.6)^ce^	*P* =.002
Working Memory **	107.0 (13.6)^bc^	104.5 (16.7)^de^	94.1 (14.99)^bd^	93.0 13.4)^ce^	*P* =.001
Trail Making Test‐B	61.7 (28.7)^c^	67.7 (27.9)^e^	91.7 (60.5)	87.3 (28.6)^ce^	*P* =.006
Stroop‐CW^†^	111.7 (22.3)^c^	106.0 (24.8)^de^	98.2 (24.8)^bd^	86.6 (21.6)^ce^	*P* =.003
WCST‐64 PR^‡^	9.4 (6.2)^c^	9.7 (5.1)^e^	10.1 (5.7)^f^	15.7 (13.96)^cef^	*P* =.04

Note

For each of the cognitive measures, means with similar superscripts represent significant pairwise differences between groups at *P* <.05 (Sidak correction). Numbers in parentheses are standard deviations.

* From the Weschler Adult Intelligence Scale‐III.

** From the Weschler Memory Scale‐III.

^†^ From the Color‐Word Interference trial of the Stroop Test.

^‡^ Number of perseverative responses from the Wisconsin Cart Sort Test‐64.

MANOVA was significant, Hotelling's T=0.85, F = 4.1, *df*=33,476, *P* <.001. Significant (*P* < .001) univariate effects were obtained for all measures (*P* < .001) with the exception of WSCT‐PR (*P* = .001).

Post hoc pairwise comparisons showed Cluster 1 to perform worse than controls only on memory metrics including Auditory Delayed (*P *= .014), Visual Immediate (*P *= .049), and Visual Delayed (*P *= .036) indices. Cluster 2 performed worse than controls across all cognitive measures except WCST‐PR (*P *= .74) and Auditory Immediate memory (*P *= .09). Cluster 3 performed worse than controls across all measures with all *P*'s < .001 except for WCST‐PR (*P *= .016), Trails‐B (*P* = .03), and Visual Immediate (*P *= .001). Additional cross‐cluster comparisons demonstrated significant differences between Cluster 1 and Cluster 2 on all measures except Trails‐B and WCST, and significant differences between Cluster 1 and 3 on all measures except Visual Immediate memory. Cluster 2 differed from Cluster 3 only on the WCST‐PR and Auditory Delayed memory.

### 3.4 Clinical seizure and sociodemographic variables

There were no significant relationships between cluster membership and age (*P* = .81), gender (*P* = .22), handedness (*P* = .94), employment status (*P* = .14), marital status (*P* = .39), or current financial assistance (*P* = .14). Education was associated with cluster membership (*P* = .001) with lower education associated with increasing behavioral risk (13.8, 12.9, and 11.5 years of education across Clusters 1‐3). Regarding clinical epilepsy characteristics, there were no associations between behavioral phenotype groups and age of onset (*P* = .95), duration of epilepsy (*P* = .58), number of medications (*P* = .12), time since last seizure (*P* = .81), and estimated number of lifetime generalized seizures (*P* = .37). There was also no relationship between behavioral phenotype and the frequency of simple (*P* = .896), complex (*P* = .79), or secondarily generalized seizures (*P* = .52) during the past year, the frequency of all seizures combined (0.89) over the past year, nor the nature of change in seizure frequency compared to the prior year (*P* = .24) (See Table S1 for details across clusters).

Regarding patient‐rated seizure severity, pairwise comparisons showed Cluster 1 to report less severity than Clusters 2 (*P *= .002) and 3 (<0.001) with no difference between Clusters 2 and 3 (*P *= .71). Examining TLE patients who underwent ictal EEG monitoring (n = 55), there was no relationship between cluster membership and left (n = 24) versus right (n = 20) onset (*P* = .32), but a significant relationship between unilateral (n = 44) versus bilateral onset (n = 10) onset (*P* = .015) with the majority of the bilateral group [80%] in Clusters 2 or 3% vs 48% of the unilateral group.

### 3.5 Psychiatric and quality‐of‐life status

Table 3 shows significant associations between group and the presence of a current Axis 1 disorder (*X* ^2^=14.3, *df*=3, *P* = .02), current mood diagnosis (*X* ^2^=34.1, *df*=9, *P *= <.001), current treatment with psychiatric medication (*X* ^2^=12.6, *df*=3, *P* = .005), and lifetime‐to‐date Axis 1 disorder (*X* ^2^=8.35, *df*=3, *P* = .034). There was no relationship between cluster group and family psychiatric history (*P* = .20) or history of depression in a first‐degree relative (*P* = .30). Examining the distribution of specific disorders across TLE clusters, the rate of current anxiety disorders (5.7%, 20%, 27%) and adjustment disorders (8.6%, 3.3%, 16%) were highest in Cluster 3, as was the proportion of TLE patents with >1 current psychiatric diagnosis (2.8%, 6.6%, 11%).

**TABLE 3:** Psychiatric and patient self‐report results

	Controls	Cluster 1	Cluster 2	Cluster 3
Current Axis 1 Dx	22%	17%	47%	56%
Current Mood Dx	0%	0%	23%	22%
Current Psychiatric Treatment	6%	9%	36%	33%
LTD Axis 1	48%	45%	74%	72%
BDI‐II	4.7 (5.1)	4.4 (4.6)	8.1 (4.7)	18.6 (8.1)
QOL‐89 Total	na	56.2 (5.2)	48.5 (6.9)	39.5 (7.97)
LSS Total	na	39.9 (6.9)	44.6 (8.6)	45.6 (9.6)

Note

Numbers in parentheses are standard deviations.

Abbreviations: BDI‐II, Beck Depression Inventory; DX, diagnosis; LSS, Liverpool Seizure Severity Scale; LTD, lifetime to date; QOL, Quality of Life in Epilepsy (89‐item).

ANOVA for the Beck Depression Inventory‐II was significant, F = 48.3, *df*=3, *P* < .001. Pairwise comparisons showed no significant difference between controls and Cluster 1, but controls had significantly lower BDI scores compared to Cluster 2 (*P* = .022) and Cluster 3 (*P* < .001); Cluster 1 was significantly lower than Clusters 2 (*P* =.034) and 3 (*P *= <.001), and Cluster 2 was lower than Cluster 3 (*P* < .001).

Regarding quality of life in the epilepsy groups, ANOVA was significant, F = 18.7, *df*=2, *P* < .01. Pairwise comparisons showed Cluster 1 to report significantly better QOL than Cluster 2 (*P *= .002) and Cluster 3 (<.001). Cluster 2 reported significantly better QOL than Cluster 3 (*P* = .001) (Table 3).

### 3.6 Cortical thickness and subcortical volumes

Overall, there was no exacerbation of regional abnormalities in cortical thickness and volume, or subcortical and cerebellar regions of interest, moving across the behavioral phenotype groups (Figure S3). Regarding cortical thickness, compared to controls there were differences in left precuneus (Cluster 1) or no differences (Cluster 2 and Cluster 3). Regarding cortical volume, compared to controls there were differences in left insula, superior temporal, supramarginal and right precuneus, insula, temporal pole, and superior parietal regions (Cluster 1), no differences (Cluster 2), or left inferior and middle temporal regions (Cluster 3). Similarly, these was no pattern of increasing abnormality in subcortical regions of interest with cluster membership.

## 4 DISCUSSION

Key findings from this investigation include the following. First, as expected, patients with TLE *as a group* exhibited significantly elevated symptoms of emotional‐behavioral distress compared to controls across all SCL‐90‐R scales. In that context, cluster analysis identified distinct latent groups, or behavioral phenotypes, that varied systematically in their SCL‐90‐R symptom profiles (ie no, mild‐to‐moderately abnormal, markedly abnormal). Second, clinical epilepsy (patient‐perceived seizure severity, bitemporal seizure onset) and sociodemographic (education) correlates of phenotype membership were identified. Third, behavioral phenotypes were significantly associated with neuropsychological abnormalities indicating substantial multimorbidity between these important functional domains. Fourth, concurrent validity of the behavioral phenotypes was reflected in orderly relationships with the presence of current and lifetime‐to‐date Axis 1 diagnoses including mood disorder and current medication treatment for a mental health condition. Self‐report measures indicated stepwise declining quality of life and increasing severity of depression symptoms from Cluster 1 to Cluster 3. Finally, the latent groups exhibited minimal systematic abnormalities in cortical thickness and volume.

### 4.1 Behavioral phenotypes

Using the SCL‐90‐R, patients with TLE exhibited more abnormal scores across all nine behavior problem scales, findings that are entirely consistent with the long‐appreciated pattern of elevated psychopathology in epilepsy generally, and TLE in particular.^41^, ^42^ But as shown to be the case in regard to cognition,^9^, ^10^ these modal profiles are arguably misleading as there is significant heterogeneity across individual patients which was captured through the application of an unsupervised machine learning approach that revealed three distinct latent groups or behavioral phenotypes. The largest group (Cluster 1, 42% of TLE cohort) was completely within the SCL‐90‐R average range with no behavioral problem scales elevated compared to controls, in fact exhibiting several scales significantly lower (better) than controls. The second group (Cluster 2, 35% of TLE cohort) exhibited elevated scores across all scales compared to both the controls and Cluster 1, while the third group (Cluster 3, 23% of TLE cohort) exhibited the most abnormality across all scales compared to controls as well as Clusters 1 and 2, with four of the scale elevations falling in the pathognomonic range. Thus, a spectrum of SCL‐90‐R profiles was reflected in these latent groups.

The behavioral phenotype groups identified here were characterized predominantly by progressive increases in psychopathology as opposed to specific psychopathological features. In the larger epilepsy neurobehavioral phenotype literature, the patterns shown here have been observed in cross‐sectional and/or prospective investigations of quality of life,^43^, ^44^ cognitive trajectories of children treated surgically or medically,^45^ and most patterns of depression in mothers of children with new onset epilepsy.^46^ Cognitive phenotype investigations of adults with temporal lobe epilepsy have more typically identified varying patterns of abnormality across groups ^9^, ^10^, ^11^, ^13^, ^14^, ^16^, ^17^ as well as in patterns of executive function in children with epilepsy (Modi et al, 2019). That said, as this is the first attempt to characterize behavioral phenotype groups in temporal lobe epilepsy, more research is needed using other behavioral metrics and extension to other epilepsy syndromes to derive a fuller understanding of the taxonomy of behavioral abnormality in epilepsy and its associated patterns.

### 4.2 Clinical and sociodemographic correlates of behavioral phenotypes

Associations of cluster membership with sociodemographic factors were modest with decreasing years of education linked to increasing behavioral risk. Relationships between the behavioral phenotypes and clinical epilepsy variables occurred in relation to patient‐reported seizure severity and bitemporal as opposed to unilateral ictal seizure onset, although the latter finding is tempered by the modest sample size of the bitemporal group, hence in need of replication. There was no relationship between unilateral left versus right temporal lobe onset and cluster membership, a not unexpected finding.^47^ While a suggestion of more severe epilepsy in Clusters 2 and 3 is suggested by these findings, no other clinical relationships were detected (ie, age of onset, duration of epilepsy, time since most recent seizure, estimated lifetime generalized seizures, number of ASMs).

### 4.3 Neuropsychological correlates of behavioral phenotypes

There were orderly cognitive differences across the phenotypes with Cluster 1 comparable to controls across metrics of intelligence and executive function with differences limited to verbal and visual memory, representing classic cognitive consequence of TLE.^48^ Clusters 2 and 3 exhibited greater cognitive abnormalities across a broader range of cognitive domains with the anomalies greatest in Cluster 3 patients, impacting intelligence, memory, and executive function. Similar findings among between behavioral phenotypes and cognition in children with new/recent onset epilepsies where, again, there was also a stepwise association with worsening cognition linked to worsening behavioral phenotypes.^49^ That behavioral and cognitive abnormalities can “travel together” is an important indication of so‐called multimorbidity,^12^, ^19^ the direction of causality in established epilepsy difficult to discern, but the presence of this relationship among youth with new onset epilepsies raises the possibility that both comorbidities may be related to a common but yet to be identified etiology^42^, ^50^, ^51^ which clearly demands additional investigation.

### 4.4 Psychiatric correlates of behavioral phenotypes

As the SCL‐90‐R is a self‐report measure an important question is the degree to which the behavioral phenotypes track with external gold standard measures such as structured psychiatric interviews and diagnostics, that is, is there evidence of concurrent validity? In this regard, there were significant relationships between the behavioral clusters and the rate of current and lifetime‐to‐date Axis 1 diagnoses as well as history of mental health treatment. There was a sharp difference between Cluster 1 and Clusters 2 and 3, with more modest diagnostic differences between Clusters 2 and 3, but with consistently worse status in Cluster 3, including the highest rate of anxiety and adjustment disorders as well proportion of patients with >1 current psychiatric diagnosis. Further, health‐related quality of life showed a significant stepwise decline from Cluster 1 through Cluster 3 and a corresponding stepwise increase in total Beck Depression Inventory scores with Cluster 3 the most clinically impacted.

At the level of analysis presented in the paper, the behavioral clusters were derived through simultaneous consideration of all SCL‐90‐R scales. This behavioral phenotyping process is suboptimal for linking specific Axis 1 diagnoses with clinical elevations on specific SCL‐90‐R scales. Such an analysis would require phenotyping patients through an actuarial process (eg identify patients with singular abnormality on specific behavior problem scales (eg depression)) or specific combinations of scales (eg depression and anxiety) and then relating those phenotypes to Axis 1 diagnoses to determine specificity. This actuarial process is well underway in the cognitive phenotype literature^11^, ^13^ and will certainly follow in regard to behavioral phenotypes. At this very early stage, the stepwise relationship between cluster membership and formal diagnostic rates is noteworthy for providing reassuring evidence of concurrent validity.

### 4.5 Imaging correlates of behavioral phenotypes

Examination of regional cortical (thickness and volume) and subcortical (volume) imaging correlates of behavioral phenotype membership was informative for the absence of relationships. Regional analyses of cortical volume and thickness have been variably linked to the cognitive phenotypes of temporal lobe epilepsy, with more consistent positive relationships reported using diffusion MRI or resting state fMRI.^9^, ^11^, ^13^, ^16^, ^17^ Our inability to identify stepwise abnormalities in regional analyses of cortical volume or thickness across the behavioral phenotypes should be addressed in future research with larger sample sizes, more contemporary imaging platforms, alternative analytics (eg network science), and other neuroimaging approaches that are more sensitive to detecting network abnormalities.

### 4.6 Implications of behavioral phenotypes

Identification of behavioral phenotypes could be of theoretical and clinical import for several reasons. First, the phenotype approach offers to alter perceptions of the behavioral risk associated with the epilepsies. Conventional findings comparing mean scores of individuals with epilepsy compared to controls, such as those provided in Figure 1, are replete in the epilepsy‐behavioral literature and collectively infer substantial behavioral risk to epilepsy. The phenotype approach harnesses the underlying heterogeneity in behavioral risk and clearly identifies the majority of behaviorally unaffected persons (42%) and the minority of extremely behaviorally disordered groups (23%)—a more accurate depiction of behavioral risk in general and a perspective which is reflective of the “spectrum of risk” perspective. Second, while the field tends to focus on individual comorbidities (eg cognition, behavior, quality of life, learning disorders), this phenotype approach along with characterization of its correlates points to the substantial *multimorbidity* that exists. Here, the higher behavioral risk group had substantially more comorbid cognitive disorder, impaired quality of life, and arguably more formal psychiatric disorder, all of which points to the substantial treatment challenges that may be linked to distinct phenotype groups. Third, and relatedly, the mechanisms underlying this identified multimorbidity are important to understand. Is it causally related, for instance does severe psychopathology lead to compromised cognition, the implication then being that behavioral treatment may benefit cognition, or are both related to a third independent factor (eg genetic, neurobiological, social). The very presence of this multimorbidity and its underlying mechanisms(s) represent important research areas going forward. Finally, the development of efficient approaches for identification of phenotype membership would contribute clinically to timely identification and initiation of needed intervention(s).

### 4.7 Limitations and future directions

This cross‐sectional investigation focused on a commonly used measure of self‐reported emotional‐behavioral distress (SCL‐90‐R) that was subjected to cluster analysis to derive the behavioral phenotype groups. This is a measure that continues to be used frequently in international epilepsy research (eg 21, 23, 52, 53), but one that is known to have limitations, particularly when used in isolation (eg see [54, 55], for a critical review for and investigation of the SCL‐90‐R in neurological patients). Careful use and interpretation of psychopathology measures not normed on general medical and neurological patients is an important concern and especially critical is multimodal assessment (eg concurrent psychiatric and other neurobehavioral assessment) to confirm that self‐report inventories yield results that are actually reflective of psychiatric risk^55^ as was done in the current study. But many other self‐report and proxy‐based measures of psychopathology exist and the generalizability of the current findings to those measures remains to be determined, as well as the concurrent validity of any obtained taxonomies. The important issue of the relative value of any particular behavioral measure in the identification of phenotypes could be addressed by a comparative analysis of clustering profiles and the specifics of their concurrent and prospective validation contrasting several different behavioral measures in the same epilepsy population—an important task for the future.

Furthermore, the underlying etiology and prospective course of identified behavioral phenotypes remain critical issues for future research including the application of more sophisticated imaging of potential underlying neurobiological abnormalities with a focus on disruptions of networks integral for emotional‐behavioral status.^56^ Also important will be the pursuit of phenotypic membership with an actuarial approach, one that focusses on classification of *individual* patients with isolated impairments as has been undertaken in cognitive research.^11^, ^13^ Finally, the degree to which these behavioral phenotypes generalize to other epilepsy syndromes versus require syndrome‐specific modifications will help to inform the relationship of the evolving taxonomy of the neurobehavioral comorbidities of epilepsy to the classification of the epilepsies.

## CONFLICT OF INTEREST

The authors have no significant relationship with, or financial interest in, any commercial companies pertaining to this article.

## ETHICAL APPROVAL

We confirm that we have read the Journal's position on issues involved in ethical publication and affirm that this report is consistent with those guidelines.

## Supporting information

Fig S1

Fig S2

Fig S3

Table S1

Supplementary Material
