# Supplementary figures and images for: Behavioral phenotypes of temporal lobe epilepsy
Source: Epilepsia Open. 2021 May 5;6(2):369–80. doi: 10.1002/epi4.12488 (PMC8166791; doi:10.1002/epi4.12488)

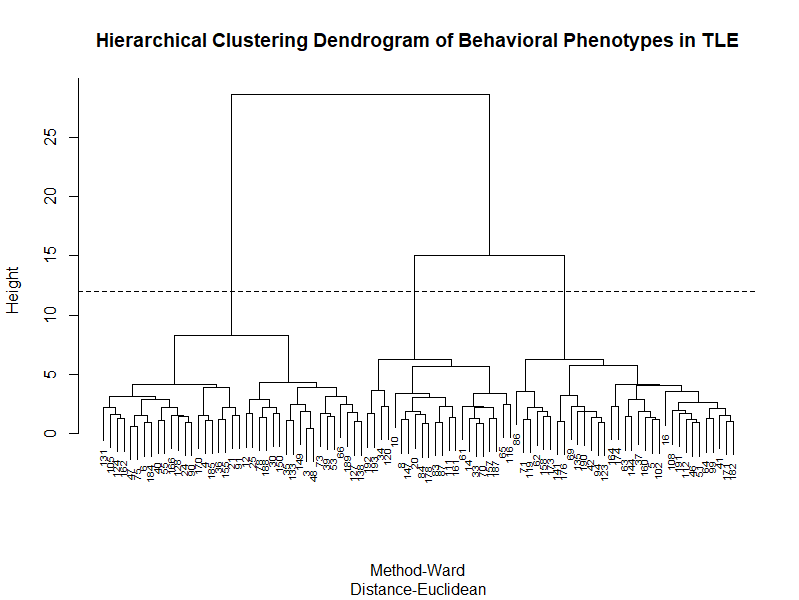

Supplement: Supplementary file 1 — Fig S1 [file EPI4-6-369-s003.tiff]

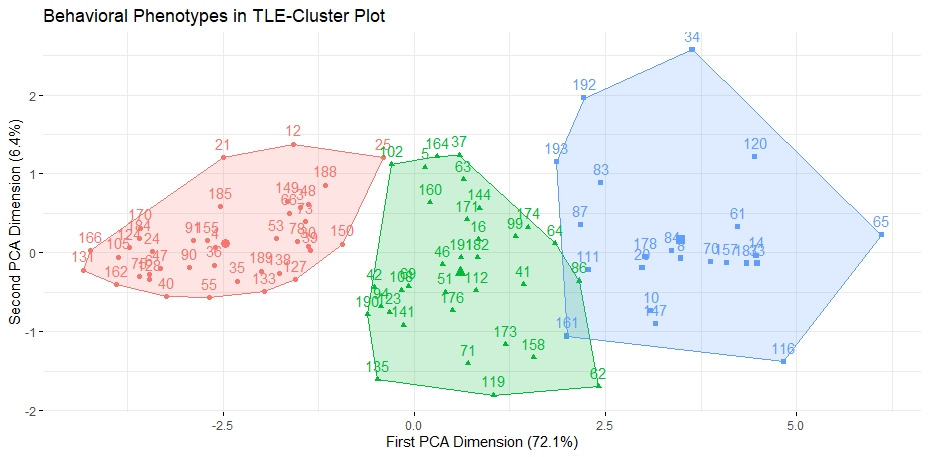

Supplement: Supplementary file 2 — Fig S2 [file EPI4-6-369-s002.tiff]
