# Supplementary material for: Behavioral phenotypes of temporal lobe epilepsy
Source: Epilepsia Open. 2021 May 5;6(2):369–80. doi: 10.1002/epi4.12488 (PMC8166791; doi:10.1002/epi4.12488)

**Supplemental Material 3**

Cold colors are regions where a cluster group metric is abnormal (thinner cortex or decreased volume) compared to control subjects. The results are cluster corrected such that each contiguous region has a corrected p-value < 0.05. Displayed are the log p-value of the group difference (Control versus Cluster group) at each vertex on the cortical surface.

***Cortical Thickness***

**Cluster 1 Thickness**

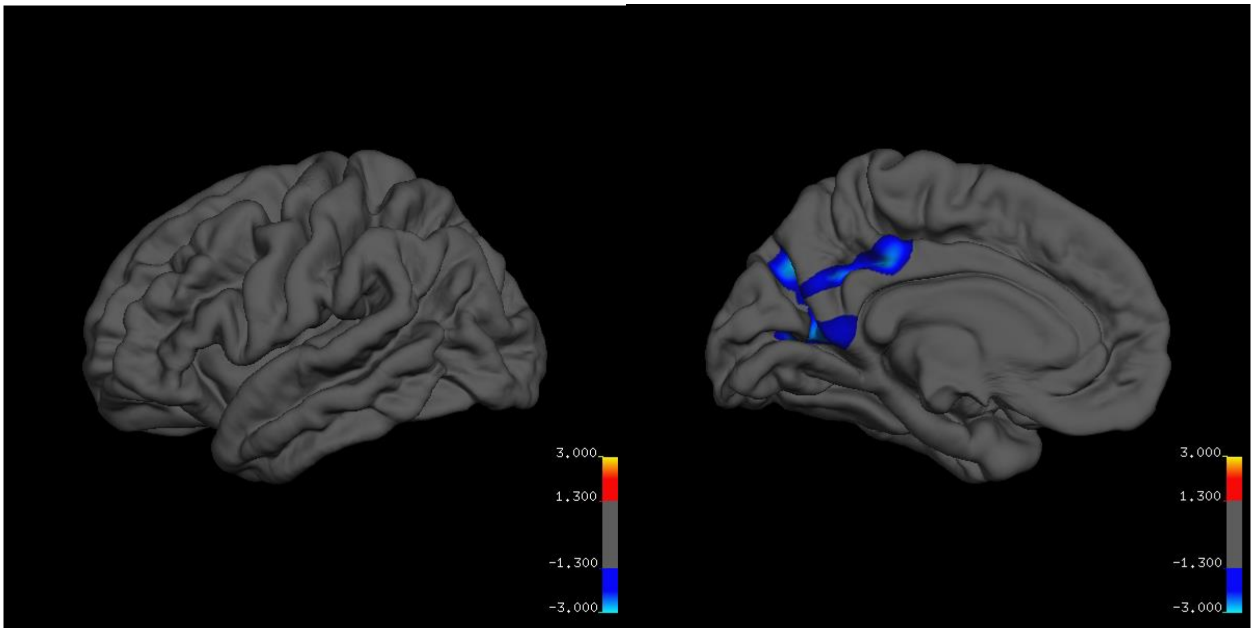

***Cortical Volume***

## Cluster 1 Volume

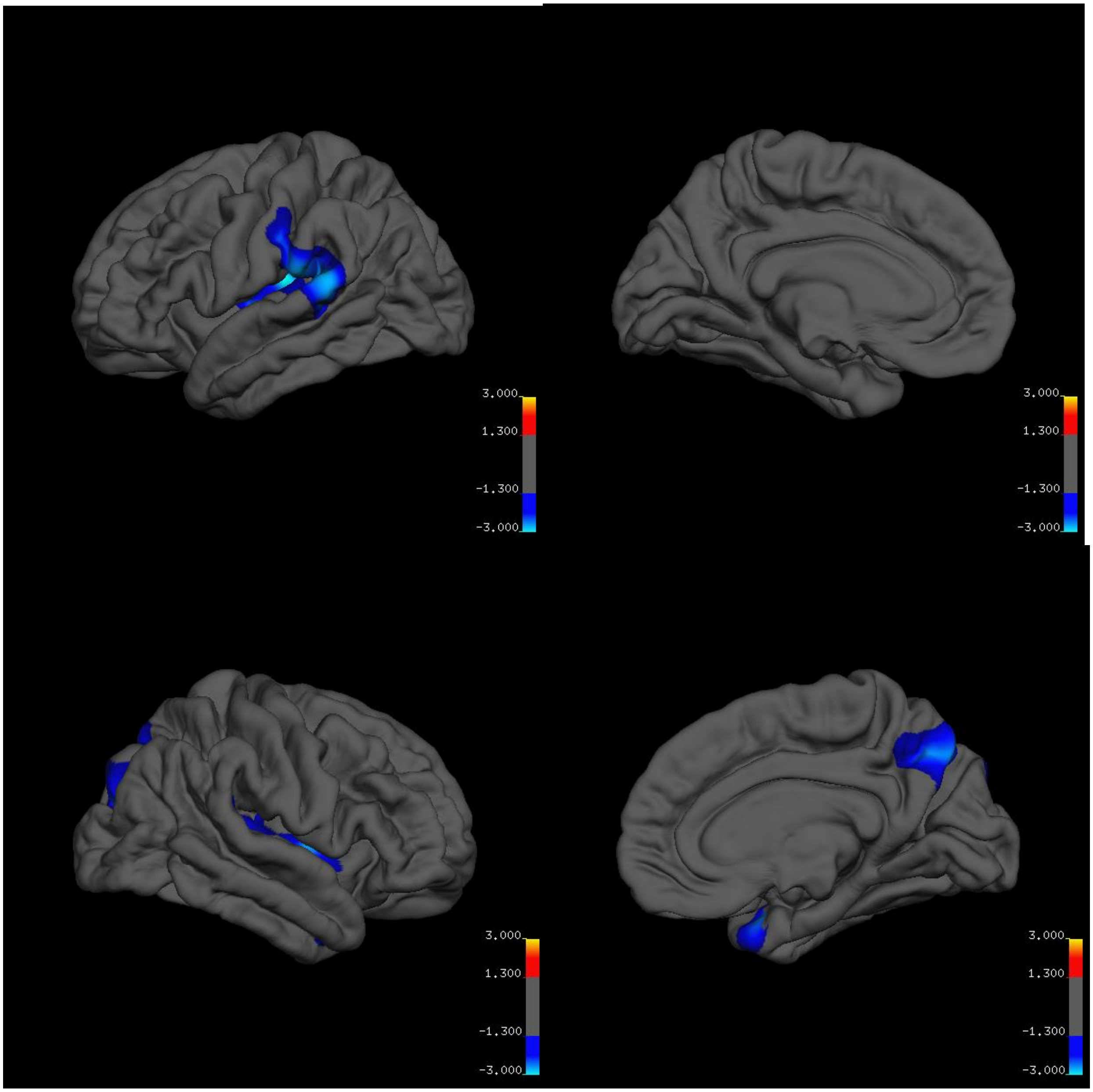

**Cluster 3 Volume**

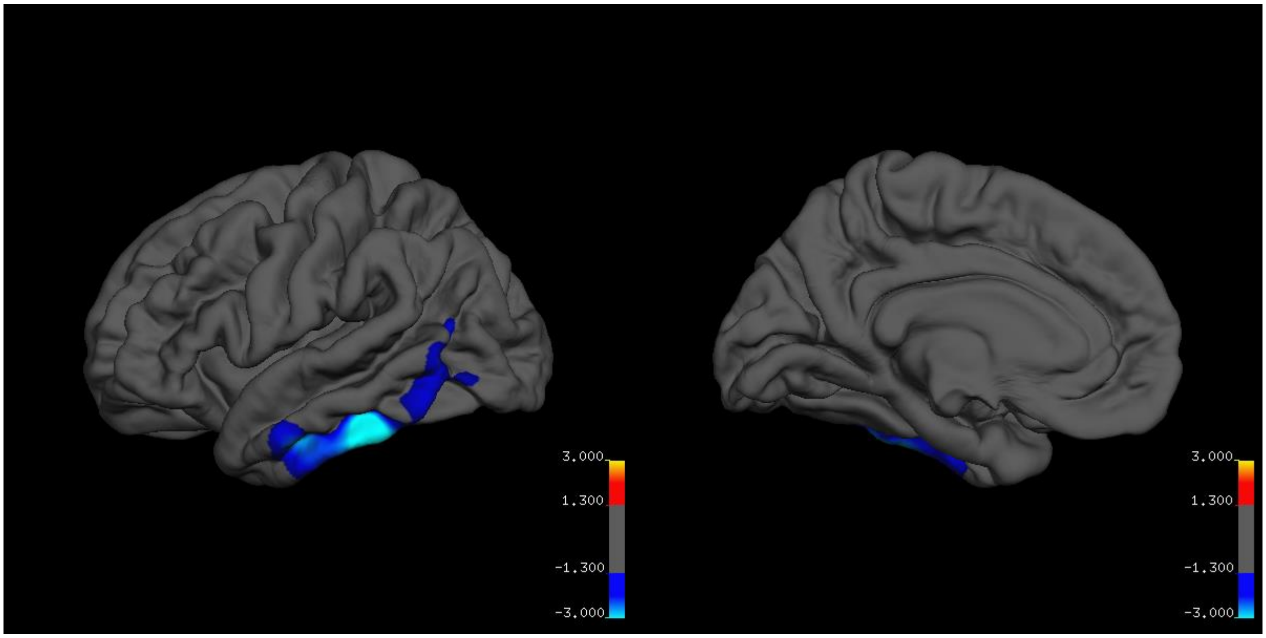

Supplement: Supplementary file 3 — Fig S3 [file EPI4-6-369-s001.pdf]
