# Supplementary material for: Behavioral phenotypes of temporal lobe epilepsy
Source: Epilepsia Open. 2021 May 5;6(2):369–80. doi: 10.1002/epi4.12488 (PMC8166791; doi:10.1002/epi4.12488)
Supplement: Supplementary file 4 — Table S1 [file EPI4-6-369-s004.docx]

**Distribution of Seizure Variables Across Clusters**

Estimated Frequency of Simple Partial Seizures the Past Year

| Cluster | None | Daily | Weekly | Monthly | Yearly | Unsure |
| --- | --- | --- | --- | --- | --- | --- |
| 1 | 30.8% | 5.1% | 15.4% | 30.8% | 15.4% | 2.6% |
| 2 | 33.3% | 6.1% | 21.2% | 18.2% | 15.2% | 6.1% |
| 3 | 31.8% | 13.6% | 13.6% | 22.7% | 9.1% | 9.1% |

Estimated Frequency of Complex Partial Seizures Past Year

| Cluster | None | Daily | Weekly | Monthly | Yearly | Unsure |
| --- | --- | --- | --- | --- | --- | --- |
| 1 | 23.1% | 0% | 23.2% | 35.9% | 12.8% | 5.1% |
| 2 | 15.2% | 0% | 21.2% | 39.4% | 18.2% | 6.1% |
| 3 | 18.2% | 4.5% | 22.7% | 40.9% | 4.5% | 9.1% |

Estimated Frequency of Secondarily Generalized Seizures the Past Year

| Cluster | None | Daily | Weekly | Monthly | Yearly | Unsure |
| --- | --- | --- | --- | --- | --- | --- |
| 1 | 74.4% | 0% | 2.6% | 15.4% | 7.7% | 5.1% |
| 2 | 75.8% | 3.0% | 3.0% | 9.1% | 9.1% | 6.1% |
| 3 | 59.1% | 0% | 13.6% | 18.2% | 9.1% | 9.1% |

Estimated Frequency of All Seizures the Past Year

| Cluster | None | Daily | Weekly | Monthly | Yearly |
| --- | --- | --- | --- | --- | --- |
| 1 | 12.8% | 5.1% | 20.5% | 48.7% | 5.1% |
| 2 | 15.2% | 3.0% | 24.2% | 39.4% | 6.1% |
| 3 | 9.1% | 13.6% | 27.3% | 36.4% | 9.1% |

Experience Seizure Free Intervals of at Least One Year?

| Cluster | No | Yes | Unsure |
| --- | --- | --- | --- |
| 1 | 46.2% | 46.2% | 7.7% |
| 2 | 51.5% | 36.4% | 12.1% |
| 3 | 54.5% | 40.9% | 4.5% |

Estimated Number of Lifetime Secondarily Generalized Seizures

| Cluster | 0-49 | 50-99 | 100+ |
| --- | --- | --- | --- |
| 1 | 79.6% | 15.4% | 5.1% |
| 2 | 75.7% | 12.1% | 12.1% |
| 3 | 81.8% | 13.6% | 4.5% |

Seizure Frequency This Year Compared to Past Years

| Cluster | Same | Better | Worse | Unsure |
| --- | --- | --- | --- | --- |
| 1 | 48.7% | 25.6% | 23.1% | 2.6% |
| 2 | 42.4% | 24.2% | 27.3% | 6.1% |
| 3 | 13.6% | 27.3% | 54.5% | 4.5% |
