# Supplementary material for: Behavioral phenotypes of temporal lobe epilepsy
Source: Epilepsia Open. 2021 May 5;6(2):369–80. doi: 10.1002/epi4.12488 (PMC8166791; doi:10.1002/epi4.12488)
Supplement: Supplementary file 5 — Supplementary Material [file EPI4-6-369-s005.docx]

**Supporting Information**

Supplemental figure 1. Hierarchical clustering dendrogram of behavioral phenotypes in TLE

Supplemental figure 2. Behavioral phenotypes in TLE cluster plot

Supplemental figure 3. Cortical thickness and volume differences

Supplemental table 1. Clinical seizure features across TLE clusters

**Footnote for Figures 1 and 2**

Note: Som=Somatization, LOC=Obsessive-compulsive, IS=Interpersonal sensitivity, DEP=Depression, ANX= Anxiety, HOS=Hostility, PHOB=Phobic anxiety, PAR=Paranoid ideation, PSY=Psychoticism
